# Detecting Deep Divergence in Seventeen Populations of Tea Geometrid (*Ectropis obliqua* Prout) in China by COI mtDNA and Cross-Breeding

**DOI:** 10.1371/journal.pone.0099373

**Published:** 2014-06-10

**Authors:** Gui-Hua Zhang, Zhi-Jun Yuan, Chuan-Xi Zhang, Kun-Shan Yin, Mei-Jun Tang, Hua-Wei Guo, Jian-Yu Fu, Qiang Xiao

**Affiliations:** 1 Key Laboratory of Tea Plants Biology and Resources Utilization of Agriculture Ministry, Tea Research Institute, Chinese Academy of Agricultural Sciences, Hangzhou, China; 2 Institute of Insect Science, Zhejiang University, Hangzhou, China; Australian Museum, Australia

## Abstract

The tea geometrid (*Ectropis obliqua* Prout, Lepidoptera: Geometridae) is a dominant chewing insect endemic in most tea-growing areas in China. Recently some *E. obliqua* populations have been found to be resistant to the nucleopolyhedrovirus (*Eo*NPV), a host-specific virus that has so far been found only in *E. obliqua*. Although the resistant populations are morphologically indistinguishable from susceptible populations, we conducted a nationwide collection and examined the genetic divergence in the *COI* region of the mtDNA in *E. obliqua*. Phylogenetic analyses of mtDNA in 17 populations revealed two divergent clades with genetic distance greater than 3.7% between clades and less than 0.7% within clades. Therefore, we suggest that *E. obliqua* falls into two distinct groups. Further inheritance analyses using reciprocal single-pair mating showed an abnormal F_1_ generation with an unbalanced sex ratio and the inability to produce fertile eggs (or any eggs) through F1 self-crossing. These data revealed a potential cryptic species complex with deep divergence and reproductive isolation within *E. obliqua*. Uneven distribution of the groups suggests a possible geographic effect on the divergence. Future investigations will be conducted to examine whether *Eo*NPV selection or other factors prompted the evolution of resistance.

## Introduction

Tea, *Camellia sinensis* (L.) O. Kuntze, originated from China and is now grown in almost sixty countries. The diversified regional climate, planting history, tea varieties, cultivation management, pest management model, tea production characteristics and environmental factors affect the distribution of insects in tea plantations worldwide. China, the largest tea growing country and with a long history of tea planting, processing and consumption, has more than 2,600 cultivars and six major categories of tea across three climatic zones and four tea ecological areas. Consequently, the pest fauna in tea fields is significantly diversified.

Loopers or inchworm moths (Lepidoptera: Geometridae) are one of the largest insect families, composed of nearly 23,000 species worldwide [1]–[3]. The tea geometrid (*Ectropis obliqua* Prout, Lepidoptera: Geometridae) was named by Prout in the year of 1915 [4]–[6]. *E. obliqua* has 4–5 generations a year in most areas, and it is now the most important chewing pest in tea plantations in China. This pest infests thousands of hectares a year, especially in the lower reaches of the Yangtze River which is a key conventional green tea producing area, including Zhejiang, Jiangsu and Anhui provinces [2]. *E. obliqua*, a voracious insect that feeds on tea leaves and tender buds, severely reduces tea production in the summer and autumn, causing growth recession and further impacting the tea production of the following year. It has been known for a long time that chemical treatment is the most effective method to control *E. obliqua*. However, because tea is being accepted by more and more consumers as a healthy beverage, its safety and quality are also garnering more attention. Therefore, the massive use of chemicals is seriously affecting not only the safety of the tea but also the ecosystems and environment in which tea is grown [2], [7]. Nowadays biological control measures are widely accepted in China to produce safer (contaminant-free) or organic tea [7].

Biological control can be an environmentally sound and effective means of reducing or mitigating pests and pest effects through the use of natural enemies, and is a widely accepted method in agriculture systems worldwide. *E. obliqua* nucleopolyhedrovirus (*Eo*NPV) is an effective natural biological agent that has been successfully developed as a bio-insecticide in China for the control of the tea geometrid [7], [8]. Recently, we found that some tea geometrid populations were insensitive to the virus. To explain this unusual phenomenon, we collected tea geometrid populations in eight main tea production provinces from 2008 to 2011 and performed a feeding test with *Eo*NPV after morphological validation of the species. We found that some *E. obliqua* populations were susceptible to this host-specific virus, but others were insensitive. The virus was more than 700 times more virulent to the susceptible population from Yixing (YX) Jiangsu province compared to the insensitive population of Quzhou (QZ) Zhejiang province [8]. Initially, the development of resistance to *Eo*NPV, possibly caused by selection pressure in some populations, was considered as an explanation [9]. However, there were no heavy applications of *Eo*NPV in any of our seventeen collection sites. Because NPV is a highly host-specific virus [2], [8], the significant variations in susceptibility to *Eo*NPV in non-*Eo*NPV-selected geometrid populations indicates that the insect might have already developed genetic divergence before *Eo*NPV was used. In addition, we hypothesize that this divergence was induced by geographic, ecological or other factors, rather than *Eo*NPV selection pressure.

Examining divergence levels by means of molecular markers and inheritance analysis is the first step toward exploring a possible mechanism. Mitochondrial DNA (mtDNA) has been widely used in insect taxonomy and molecular systematics to elucidate the phylogenetic relationships of insects [10]–[12], especially among some sibling species and cryptic species that are difficult to distinguish solely by morphology [11], [13]. For example, partial cytochrome oxidase I (*COI*) sequences reveal that *Helicoverpa. armigera* and *H. zea* are genetically separate, albeit by a short distance [14], [15]. Combined *COI* and *COII* were used to distinguish three subspecies of the hemlock looper (*Lambdina fiscellaria*, Gn.) [16]. The widely distributed neotropical skipper butterfly (*Astraptes fulgerator*) was formerly recognized as at least 10 species in northwestern Costa Rica, but morphological analysis and a DNA barcoding study of *COI* showed that whilst *A. fulgerator* is a species complex the actual number of separate taxa is smaller than it has usually been thought to be in this region [17].

In this study, we collected seventeen populations from eight main tea-producing provinces of China. To reveal genetic divergence among the populations, phylogenetic analysis of the mtDNA *COI* sequences was conducted. Single-pair cross analysis was also conducted to verify any species-level diversification and to understand the biological effects of divergence among populations.

## Materials and Methods

### Ethics Statement

No specific permits were required for this study. *E. obliqua* is an agricultural pest and is not an endangered or protected species. All samples were collected in open tea plantations and not from any national parks or protected areas.

### Sample collection and preparation

**Figure 1:**
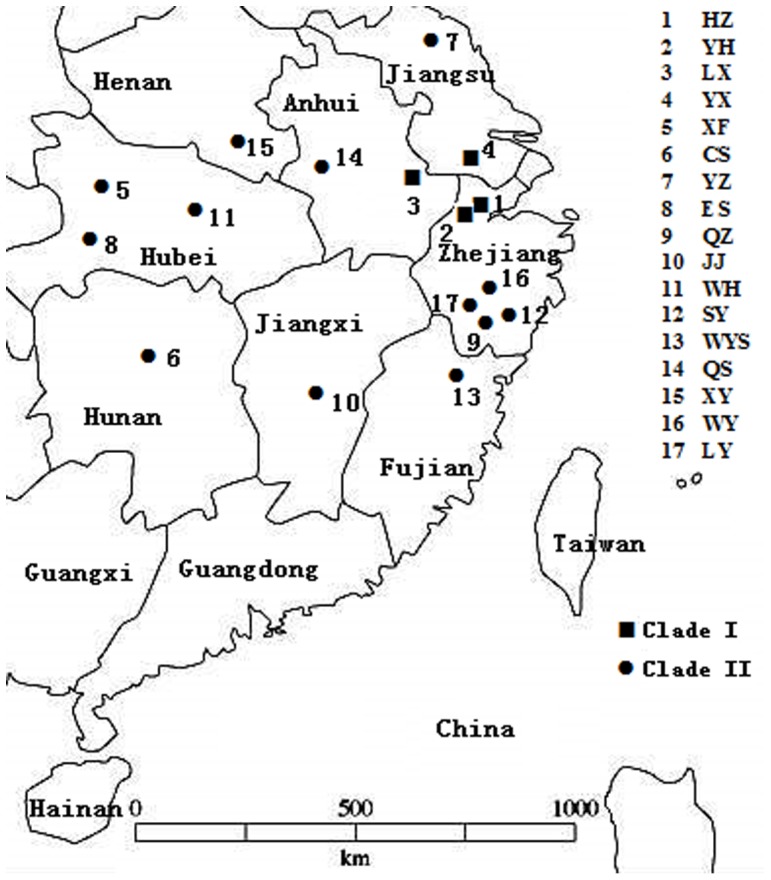
The location of sampled *E. obliqua* populations. Four populations of Clade I (square) were sampled from Hangzhou (HZ), Yuhang (YH), Langxi (LX) and Yixing (YX); thirteen populations of Clade II (closed circle) were sampled from Xiangfan (XF), Changsha (CS), Yangzhou (YZ), Enshi (ES), Quzhou (QZ), Jiujiang (JJ), Wuhan (WH), Songyang (SY), Wuyishan (WYS), Qianshan (QS), Xinyang (XY), Wuyi (WY) and Longyou (LY) in Chinese tea production areas.

*E. obliqua* specimens were collected by sweep netting from eight major tea producing provinces: Zhejiang, Anhui, Jiangsu, Jiangxi, Hubei, Fujian, Henan and Hunan, located in southern China (Figure 1). Two outgroup species of *Buzura suppressaria* Guenee and *Scopula subpunctaria* Herrich-Schaeffer were collected from a tea plantation in Hangzhou, Zhejiang province. All insect larvae were collected in summer in 2011 and 2012, rinsed in 70% ethanol three times, and stored at −80°C in 100% ethanol until DNA was extracted. All insect specimens were first identified under a stereo microscope following the current keys based on characters such as antennae, forewing and venation before further analysis [2], [5], [6]. The insects and DNA samples were deposited in the Tea Research Institute, Chinese Academy of Agricultural Sciences, Hangzhou, China.

### DNA extraction, amplification and sequencing

A total of 171 specimens were sequenced for this study, representing 17 different populations (Table 1). Total DNA was extracted from individual larvae after air-drying for 20 min. The specimen tissue was first ground with a flame-sealed pipette tip in a 1.5 ml microcentrifuge tube, and then, DNA from each individual was extracted using the DNeasy Tissue kit (Qiagen). All genomic DNA was dissolved in 100 µl deionized and sterilized water and stored at −80°C until use. Polymerase chain reactions were carried out on an ABI Veriti thermocycler in a 50 µl total volume containing 5 ng DNA, 1.5 mM MgCl_2_, 0.25 mM dNTPs, 0.2 µM of each primer and 0.5 units *taq* NEB DNA polymerase. We amplified the *COI* genes with primers 5′-GGTCAACAAATCATAAAGATATTG-3′ and 5′-TAAACTTCAGGGTGACCAAAAAAT-3′ [18]. The typical amplification profile for *COI* was melting at 94°C for 4 min, followed by 35 cycles of melting at 94°C for 30 sec, annealing at 50°C for 1 min, extension at 72°C for 1 min, followed by a final extension at 72°C for 10 min. The PCR products were detected by electrophoresis on a 1.2% agarose gel stained with ethidium bromide, purified with the Gel DNA extraction kit (Axygen), and introduced into pGEM T-easy vector (Promega). Three clones for each product were sequenced bidirectionally by an ABI 3730 automated sequencer (ABI) with the universal primers M13F: 5′-CGCCAGGGTTTTCCCAGTCACGAC-3′ and M13R: 5′-AGCGGATAACAATTTCACACAGGA-3′.

**Table 1:** Samples and collection information used in this study.

Populations	Code	Collecting locality	Longitude°(E)/Latitude°(N)	Collecting date (M/Y)	Clade (I/II)
Hangzhou	HZ	Hangzhou/Zhejiang	120.10/30.16	6/2011	I
Yuhang	YH	Yuhang/Zhejiang	119.90/30.39	5/2012	I
Langxi	LX	Langxi/Anhui	119.13/30.98	6/2011	I
Yixing	YX	Yixing/Jiangsu	119.67/31.34	6/2011	I
Quzhou	QZ	Quzhou/Zhejiang	118.88/28.97	6/2011	II
Songyang	SY	Songyang/Zhejiang	119.42/28.49	5/2012	II
Longyou	LY	Longyou/Zhejiang	119.17/29.03	5/2011	II
Wuyi	WY	Wuyi/Zhejiang	119.85/28.96	5/2012	II
Qianshan	QS	Qianshan/Anhui	116.57/30.64	5/2011	II
Yangzhou	YZ	Yangzhou/Jiangsu	119.26/32.23	5/2011	II
Wuhan	WH	Wuhan/Hubei	114.31/30.55	5/2012	II
Xiangfan	XF	Xiangfan/Hubei	112.13/32.02	5/2012	II
Enshi	ES	Enshi/Hubei	109.42/29.89	5/2012	II
Xinyang	XY	Xinyang/Henan	114.08/32.12	6/2012	II
Wuyishan	WYS	Wuyishan/Fujian	117.09/27.07	6/2012	II
Jiujiang	JJ	Jiujiang/Jiangxi	115.99/29.55	6/2012	II
Changsha	CS	Changsha/Hunan	113.26/28.27	5/2011	II

### Genetic diversity and phylogenetic analysis

Genetic diversity was assessed using DnaSP v5.10 to estimate haplotype (*Hd*) diversity [19], and the outgroup species (*B. suppressaria* and *S. subpunctaria*) were excluded from these analyses. Phylogenetic tree searches were conducted using neighbor-joining (NJ) and maximum-parsimony (MP) methods by MEGA 5 program with 1000 bootstrap replications. The software MEGA 5 was also used to estimate mean sequence divergence among populations based on the Kimura 2-Parameter (K2-P) model [20]. The software Arlequin 3.11 was used to analyze genetic differentiation [21], gene flow and molecular variance between populations. The evolutionary network was constructed via a median joining method as implemented in the Network 4.6.0.0 software.

### Propagation of Populations

All the tea geometrid populations used were first inspected using a microscope for morphological analysis. Taxonomic keys including antennae, forewing, wing, and genitalia characters were used for diagnosing specimens as *E. obliqua* [5], [6]. All collected populations were reared under the same conditions, feeding on fresh tea leaves, in a temperature-controlled room at 24±1°C, 75% relative humidity (RH), and a 12-hr light/12-hr dark photoperiod. Three populations susceptible or insensitive to virus were randomly selected for cross-breeding and maintained at least two generations prior to the start of cross-breeding and propagated in the laboratory as Xi et al. (2011) described [8]. The male and female pupae of each population were isolated and reared for mating.

### Cross-breeding of Populations

Based on the genetic divergence and molecular phylogenetic analysis of the mitochondrial *COI* gene, the populations were divided into two distinct groups. We randomly selected several populations from each group for further crossing experiments. The populations of HZ, YH, YX and LX were classified into one group, and the other thirteen populations were classified into another group. The YH, YX, LX populations from the first group and the SY, WH, QS populations from the second group were used in cross-breeding experiments. Two pairs from the YX and LX, and QS and WH populations were crossed for inter-population and intra-group studies, and YH and SY was the cross pair for inter-group population studies.

All reciprocal crosses were conducted simultaneously and under the same laboratory conditions as the parental colonies were maintained [22], [23]. Each cross was set up between one virgin female and one male in a transparent colorless glass cage, and intra-population mating was used as the control treatment. Two pairs were used for one repeat and twelve experimental repeats were for all cross-breeding or mating tests. Each pair was observed daily to record egg number, hatching rate, survival to adult, the proportion of normal adults, and the sex-ratio. The F_1_ offspring were self-crossed using the conditions mentioned above. The self-crosses of the intra-population of the F_1_ generation were the same as for the population in the control treatment, and the eggs, hatching rate and larvae development of F_2_ were simultaneously recorded. The sex-ratios across replicates were calculated by dividing the total number of females by the overall number of males in each treatment. All the other parameters were compared amongst treatments by one-way ANOVA analysis using SPSS statistical software 21.0 and the equality of means in the same column was tested by Duncan's multiple range test.

## Results

### PCR amplifications and sequences analysis

We cloned and sequenced the *COI* gene for all 171 specimens from seventeen tea geometrid populations and 20 samples from two outgroup populations. The *COI* fragments are 658 bp in length with an A+T content of 70.6%. The sequence alignment contains 157 polymorphic sites and 115 parsimony informative sites. A total of 50 *COI* haplotypes were observed. Sequences of 50 haplotypes for *E. obliqua* and 6 haplotypes for the two outgroups of *B*. *suppressaria* and *S*. *subpunctaria* were deposited in GenBank (Accession Nos. KF748178-KF748227, KF748228-KF748230, and KF748231-KF748233). The overall haplotype diversity index (*Hd*) was 0.73. The mean nucleotide diversity index (*Pi*) and mean gene flow (*Nm*) among the 17 populations were 0.018 and 0.050 respectively, and the *Fst* index of population differentiation was 0.877. Only two haplotypes QZ1 and HZ2 were shared between populations: HZ2 was shared by 21 samples from four populations of HZ, YH, LX and YX, and QZ1 was shared by 86 samples from thirteen populations of QZ, JJ, XY, QS, CS, WH, XF, LY, YZ, SY, WY, ES and WYS. The other 48 haplotypes were each found in only one population. Among the 17 populations, the LY population had just one haplotype, and other sixteen populations had more than one haplotype. The populations with the highest haplotype diversity were WH, which had seven haplotypes, and LX, which had eight haplotypes. The mean genetic divergences (*D*) and nucleotide diversity (*Pi*) differed significantly between the two groups (Table 2). In addition, the neutrality-test values (Tajima's D, Fu and Li's D and Fu and Li's F) were all significantly less than zero.

**Table 2:** Comparison of the *COI* gene evolution of two *E. obliqua* clades.

	n	h	S	Pi	D	Tajima's D	Fu and Li's D	Fu and Li's F
Clade I	53	17	21	0.00453	−2.068547	−2.16308**	−3.22719**	−3.38123**
Clade II	118	33	36	0.00332	−2.714675	−2.71467***	−5.25702**	−5.21491**

The letters n, h, S, Pi and D are the no. of *E. obliqua* samples, no. of mitochondrial DNA haplotype, no. of segregating sites, nucleotide diversity and genetic divergence respectively.

*: *P*<0.05;

**: *P*<0.02;

***: *P*<0.01.

### Genetic distance

The genetic distances among seventeen populations were calculated using the Kimura 2-parameter model in MEGA 5 (Table 3). The genetic divergences for the *COI* genes among populations of *E. obliqua* were 0.0%–4.3%, which was a large interval. They were obviously less than those between *E. obliqua* and either outgroup, which were 10.7%–12.9%. We found that the genetic distances between each pair of populations from the group containing HZ, YH, LX and YX were 0.0%–0.3%, and those between each pair of populations from the group containing QZ, JJ, XY, QS, CS, WH, XF, LY, YZ, SY, WY, ES and WYS were 0.0%–0.7%. The pairwise distances between populations from two groups were 3.7 to 4.3%. These results showed that the seventeen *E. obliqua* populations contained two deeply divergent groups, one including the populations of HZ, YH, LX and YX, and the other including the populations of QZ, JJ, XY, QS, CS, WH, XF, LY, YZ, SY, WY, ES and WYS.

**Table 3:** The average pairwise genetic distance of the populations of *E. obliqua* based on the Kimura 2-parameter model.

Population code		1	2	3	4	5	6	7	8	9	10	11	12	13	14	15	16	17	18	19
1	HZ																			
2	YH	0.001																		
3	LX	0.003	0.002																	
4	YX	0.002	0.002	0.003																
5	XF	0.037	0.037	0.037	0.037															
6	CS	0.042	0.042	0.042	0.043	0.007														
7	YZ	0.042	0.042	0.042	0.043	0.007	0.001													
8	ES	0.042	0.042	0.042	0.043	0.007	0.001	0.001												
9	QZ	0.042	0.042	0.042	0.043	0.007	0.001	0.001	0.001											
10	JJ	0.042	0.042	0.042	0.043	0.007	0.001	0.001	0.001	0.001										
11	WH	0.043	0.043	0.043	0.043	0.007	0.001	0.002	0.002	0.002	0.002									
12	SY	0.042	0.042	0.042	0.043	0.008	0.001	0.001	0.001	0.001	0.001	0.001								
13	WYS	0.042	0.042	0.042	0.043	0.007	0.001	0.001	0.001	0.001	0.001	0.002	0.001							
14	QS	0.042	0.042	0.042	0.043	0.007	0.001	0.001	0.001	0.001	0.001	0.001	0.001	0.001						
15	XY	0.042	0.042	0.042	0.042	0.007	0.000	0.001	0.001	0.001	0.001	0.001	0.000	0.001	0.001					
16	WY	0.042	0.042	0.042	0.043	0.007	0.001	0.001	0.001	0.001	0.001	0.001	0.001	0.001	0.001	0.000				
17	LY	0.042	0.042	0.042	0.042	0.007	0.000	0.001	0.001	0.001	0.001	0.001	0.001	0.001	0.000	0.000	0.000			
18	Ss	0.107	0.107	0.109	0.108	0.107	0.107	0.107	0.107	0.107	0.107	0.108	0.107	0.107	0.107	0.107	0.107	0.107		
19	Bs	0.116	0.116	0.115	0.117	0.122	0.123	0.123	0.123	0.124	0.123	0.123	0.123	0.123	0.123	0.123	0.123	0.123	0.129	

Bs (*S. subpunctaria*) and Ss (*B. suppressaria*) are the outgroups.

### Phylogenetic analysis

**Figure 2:**
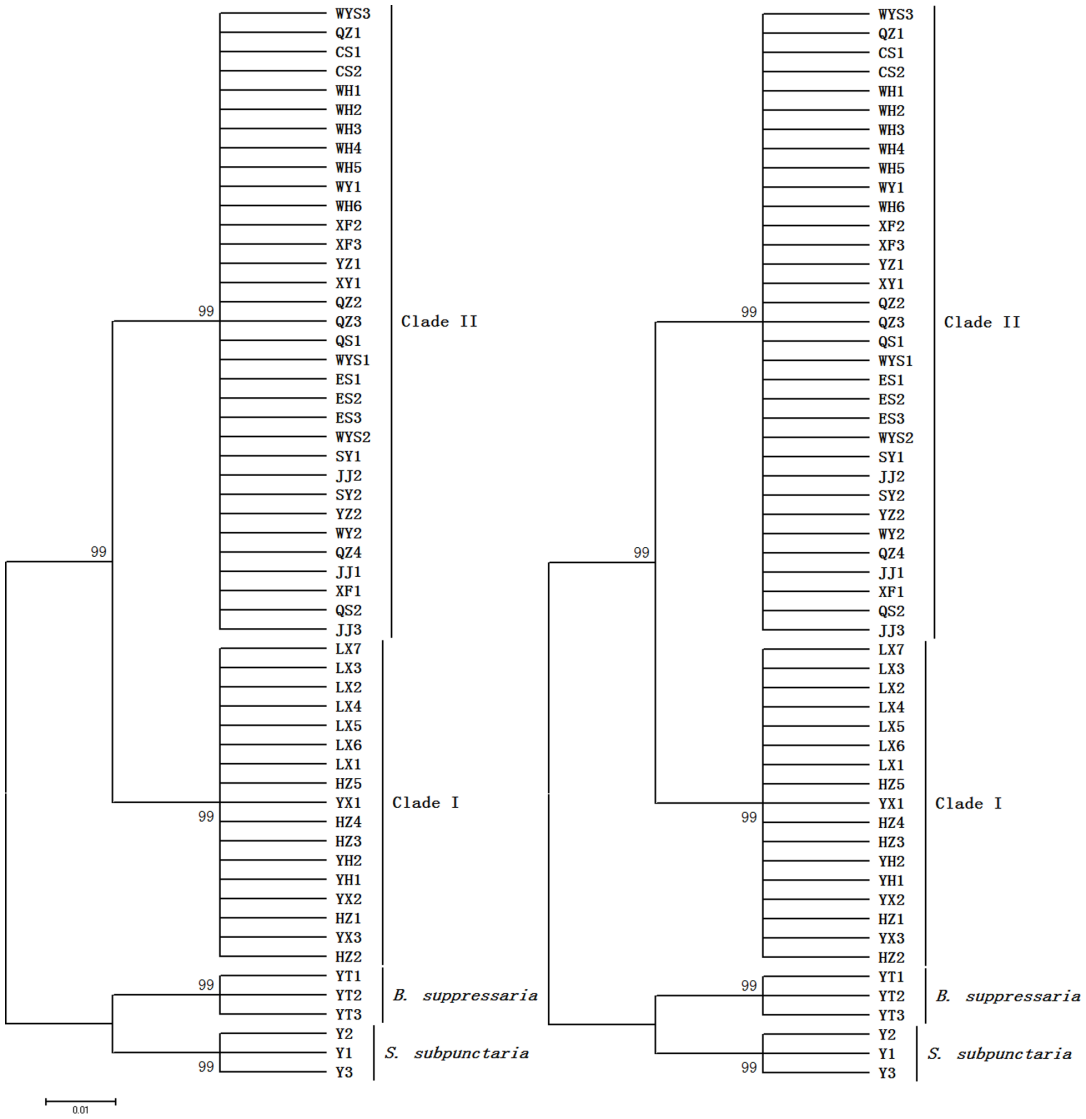
The NJ tree (L) and MP tree (R) of *E. obliqua* and two outgroup species based on *COI* sequence haplotypes. Numbers above the nodes indicate bootstrap support. The outgroups used were *B. suppressaria* and *S. subpunctaria*. The QZ1 haplotype was shared by all thirteen populations insensitive to the *Eo*NPV virus within Clade II; the HZ2 haplotype was shared by all four populations susceptible to the virus within Clade I; and other haplotypes were unique.

Based on the 50 haplotypes and two outgroups of *B. suppressaria* and *S. subpunctaria*, we analyzed the phylogenetic relationships among the seventeen populations using the NJ and MP methods in MEGA 5 (Figure 2). The phylogenetic trees were highly consistent, and both showed that all haplotypes within any population fell into a single group. The *E. obliqua* populations of HZ, YH, LX and YX clustered into one group we named Clade I, while the populations of QZ, JJ, XY, QS, CS, WH, XF, LY, YZ, SY, WY, ES and WYS clustered into another group we named Clade II. The haplotypes found in multiple populations (QZ1 and HZ2) were restricted to Clade II or Clade I populations, respectively. Because two haplotypes of QZ1 and HZ2 distributed universally within their respective clade, and the analysis of median joining network also supported they were predominant ones, they may be the original haplotypes. The phylogenetic results were consistent with the genetic distance analysis that concluded that *E. obliqua* has two deeply divergent clades in China.

### Inheritance analysis

Three populations were selected from each clade, the YH, YX and LX populations from Clade I, and the SY, WH and QS populations from Clade II. The cross of YH and SY was inter-clade, and the two pairs of YX and LX and WH and QS were intra-clade. All the crossed pairs either inter-clade or intra-clade, produced eggs. Significantly fewer eggs were observed in the cross-breeding of YH♀×SY♂ compared with the control self-cross of the SY population: the number of eggs laid per female was 357±17.31, which was less than 442±20.30 (*P*<0.01) (Table 4). All eggs produced in the intra-population and inter-population intra-clade crosses were fertile. The hatchability, survival to adult and percentage normal adults of the two cross-breeding combinations were all much lower than for intra-population self-crosses (*P*<0.01). Moreover, the F_1_ generation sex ratio (♀:♂) after cross-breeding between the YH and SY populations was highly unbalanced, at 1∶4 for the cross of YH♀×SY♂ and 1∶27 for the cross of SY♀×YH♂.

**Table 4:** The cross-breeding for the YH and SY populations of *E. obliqua*.

Cross	Replications	No. eggs laid/female	Hatchability	Survival to adult	Percentage of normal adults	Sex ratio (♀:♂)
YH♀×SY♂	12	357±15.58 B	0.01±0.00 C	0.17±0.02 B	18.23±12.25 B	1∶4
SY♀×YH♂	12	394±19.35 AB	0.54±0.05 B	0.19±0.01 B	24.70±10.06 B	1∶27
YH♀×YH♂	12	396±8.90 AB	0.74±0.09 A	0.80±0.03 A	93.49±2.05 A	1∶1
SY♀×SY♂	12	442±13.80 A	0.66±0.02 A	0.77±0.02 A	94.16±0.62 A	1∶1

Data in the table are mean ± SE. The letters following the SEs group observations in the same column that are not significantly different (*P*>0.01, Duncan's multiple range test. Means with no common letters were significantly different. Note that the values of the sex ratio (♀:♂) were not analysed in this manner.

To detect reproductive isolation level between the two *E. obliqua* clades, we conducted intra-population self-crosses of the F_1_ generation. Highly unbalanced sex-ratios and few emerged normal adults reduced the opportunities for selfcrosses and backcrosses of the F1 hybrids. We found that the YS offspring of the F_1_ generation of YH♀×SY♂ couldn't emerge within three days and therefore produced no eggs with no hatching, and the SY' offspring of the F_1_ generation of SY♀×YH♂ produced extremely few eggs with no hatching (*P*<0.01) (Table 5). Therefore, the inter-clade cross-breeding for *E. obliqua* could not produce an F_2_ generation. In addition to using the self-cross intra-population as controls, we also conducted one intra-clade cross. Compared with the self-cross controls, the F_1_ generations from cross-breeding the YX×LX populations in Clade I and the WH×QS populations in Clade II exhibited no difference in egg production, hatchability, emergence or sex ratio (Table 6, 7). Furthermore, their F_2_ and F_3_ generations also developed and produced normal offspring after self-crosses.

**Table 5:** The self-crosses of F_1_ generation from cross-breeding of the YH and SY populations.

Treatment	Replications	No. eggs laid/female	Hatchability
F_1_(YS)♀×F_1_(YS)♂	0	—	—
F_1_(SY′)♀×F_1_(SY′)♂	1	315	—
F_1_(YH)♀×F_1_(YH)♂	8	465±16.62	0.82±0.03
F_1_(SY)♀×F_1_(SY)♂	8	453±37.99	0.72±0.10

Data shown in the table are mean ± SE. There were no significant differences at the 0.01 level between mean values within columns. The YS is the F_1_ generation from cross-breeding of YH♀×SY♂, and the SY′ is the F_1_ generation from cross-breeding of SY♀×YH♂. “—” means none, that is the YS couldn't emerge within three days and therefore produced no eggs with no hatching, and the SY′ produced extremely few eggs with no hatching.

**Table 6:** The cross-breeding of the YX and LX populations within Clade I.

Treatment	Replications	No. eggs laid/female	Hatchability	Adult emergence rate	Sex ratio (♀:♂)
YX♀×LX♂	8	146±18.25	0.72±0.08	0.62±0.05	1: 1.33
LX♀×YX♂	8	172±22.65	0.68±0.14	0.56±0.01	1∶1.38
YX♀×YX♂	8	235±34.47	0.82±0.01	0.51±0.02	1∶1.57
LX♀×LX♂	8	235±54.20	0.74±0.08	0.52±0.05	1∶1.09

Data shown in the table are mean ± SE. There were no significant differences at the 0.01 level between mean values within columns. The values of sex ratio (♀:♂) were not included in this analysis.

**Table 7:** The cross-breeding of the WH and QS populations within Clade II.

Treatment	Replications	No. eggs laid/female	Hatchability	Adult emergence rate	Sex ratio (♀:♂)
WH♀×QS♂	8	115±25.47	0.54±0.04	0.95±0.04	1∶1.47
QS♀×WH♂	8	109±26.47	0.60±0.06	0.89±0.07	1∶1.54
WH♀×WH♂	8	121±17.98	0.65±0.01	0.89±0.02	1∶1.05
QS♀×QS♂	8	139±17.31	0.63±0.09	0.85±0.15	1∶1.42

Data shown in the table were mean ± SE. There were no significant differences at the 0.01 level between mean values within columns. The values of sex ratio (♀:♂) were not included in this analysis.

## Discussion

Tea is an evergreen bush with a long growing season from March to September. Growers have to spray several times to minimize herbivore damage. The development of resistance to chemical insecticides is very common in many tea insects [3], [24], possibly driven by selection pressure [9]. Although *Eo*NPV is not a chemical insecticide, resistance may still develop in target pest populations. Our previous study showed significant variations (up to 700-fold) in susceptibility to *Eo*NPV in field populations of *E. obliqua* [5], causing us to question whether resistance has evolved in this insect in a similar manner to the well-documented development of Bt resistance due to extensive use of Bt or transgenic Bt crops or to newly arising baculovirus resistance in moths [9], [25]. Therefore, we surveyed the level of *Eo*NPV used in those locations. Because the only *Eo*NPV pesticide product used in China was developed by our institute and an agricultural chemical company, we have a clear application record of this virus in tea plantation. Surprisingly, there was no record of *Eo*NPV for *E. obliqua* control in the locations of the studied populations. Hence, *Eo*NPV may not have had a direct influence on the susceptibility changes in *E. obliqua* populations. Considering the high specificity of the virus and the vast geographic variation in tea ecosystems, our second hypothesis for this study was that *E. obliqua* may have evolved deep genetic divergence to adapt to unique and localized tea ecosystems.

In this study, we first conducted phylogenetic analysis of the *COI* region of *E. obliqua* mtDNA, which is a useful tool for identifying sibling species that lack morphological characters [17], [26]–[28]. All 50 haplotypes (assembled from 171 sequences) from seventeen *E. obliqua* populations fell into two clear clades. Within a clade, populations showed close relationships, with genetic distances less than 0.7%. In contrast, the genetic divergences between clades were 3.7%–4.3%, well beyond the criterion of 2% for insect species differentiation in general or the 3% specifically suggested for lepidopteran species differentiation [29], [30]. These molecular data strongly indicated that *E. obliqua* is a cryptic complex including two deeply divergent clades. Moreover, the lack of shared haplotypes also indicated a certain degree of reproductive isolation between the clades [17].

To test species divergence and reproductive isolation, we selected three populations from each clade and conducted inter-clade crossing and F_1_ self-crossing tests. Although the number of eggs produced by inter-clade crossing was not significantly different from the in-clade self-crossed control, the egg hatchability, survival rates to adult and percentage of normal adults were significantly lower (along with an abnormal sex ratio) than those of the self-crossing controls. The selfcross of the F_1_ generation from inter-clade hybrids produced sterile eggs or no eggs, while the control group F_1_, F_2_ and F_3_ generations obtained from crossings between populations within a clade could grow, develop and produce normal progenies. The cross-breeding results further support the suggestion of reproductive isolation between two clades of *E. obliqua*.

Cryptic species complexes, virtually identical in appearance but nonetheless having reproductive isolation, are very common in nature [17], [31]–[33]. The biological characteristics of the tea plant and decreasing artificial disturbance after initial planting turn tea plantations into a shaded and stable ecosystem that is consequently a permanent habitat for herbivores. Thus, not all insects in tea plantations migrate long distances, and thus, these insects can easily colonize and develop diversified evolution in adaptive surroundings [34], [35]. Furthermore, few tea plant varieties require replacement during production due to their long life, which also decreases the genetic exchanges among *E. obliqua* populations. Many tea plantations are also located on mountains with highly diversified geography and climate, which might drive the divergence. The locations of the four populations within Clade I (HZ, YH, YX and LX) are close to each other and possess the same geographic characteristics and climate, while the Clade II locations are in the mountains with diversified geography and changeable climate within each of them. As a result, these possible factors may drive the divergent evolution in relatively isolated *E. obliqua* populations.

In summary, the detection of strong genetic divergence and reproductive isolation in *E. obliqua* provides valuable information for guiding effective control of this pest. However, more research is needed to understand the distribution of these cryptic species in tea-growing areas. Future studies also need to include clarification of the mechanism of reduced or lost sensitivity to *Eo*NPV and should explore the potential application of the reproductive incompatibility of the cryptic species for strategic pest management.
